# Crystal structure of 2-methyl­amino-3-nitro-4-*p*-tolyl­pyrano[3,2-*c*]chromen-5(4*H*)-one

**DOI:** 10.1107/S205698901500225X

**Published:** 2015-02-07

**Authors:** J. Govindaraj, Y. AaminaNaaz, Jayabal Kamalraja, Paramasivam T. Perumal, A. SubbiahPandi

**Affiliations:** a Department of Physics, Pachaiyappa’s College for Men, Kanchipuram 631 501, India; b Department of Physics, Presidency College (Autonomous), Chennai 600 005, India; c Organic Chemistry Division, Central Leather Research Institute, Adyar, Chennai 602 020, India

**Keywords:** crystal structure, pyrano[3,2-*c*]chromenone, biological activity, chromene derivatives, hydrogen bonding, crystal structure

## Abstract

In the racemic title compound, C_20_H_16_N_2_O_5_, the pyran ring adopts a shallow envelope conformation, with the stereogenic C atom displaced from the other atoms by 0.273 (2) Å. The dihedral angle between the fused-ring system and the pendant *p*-tolyl group is 87.62 (7)°. The mol­ecular conformation is consolidated by an intra­molecular N—H⋯O hydrogen bond, which generates an *S*(6) ring. In the crystal, mol­ecules are linked by C—H⋯O inter­actions, resulting in [010] chains.

## Related literature   

For background to the biological activity of chromene derivatives, see: Borges *et al.* (2005 ▸, 2009 ▸); Gibbs (2000 ▸); Varmus (2006 ▸). For a related structure, see: Narayanan *et al.* (2013 ▸).
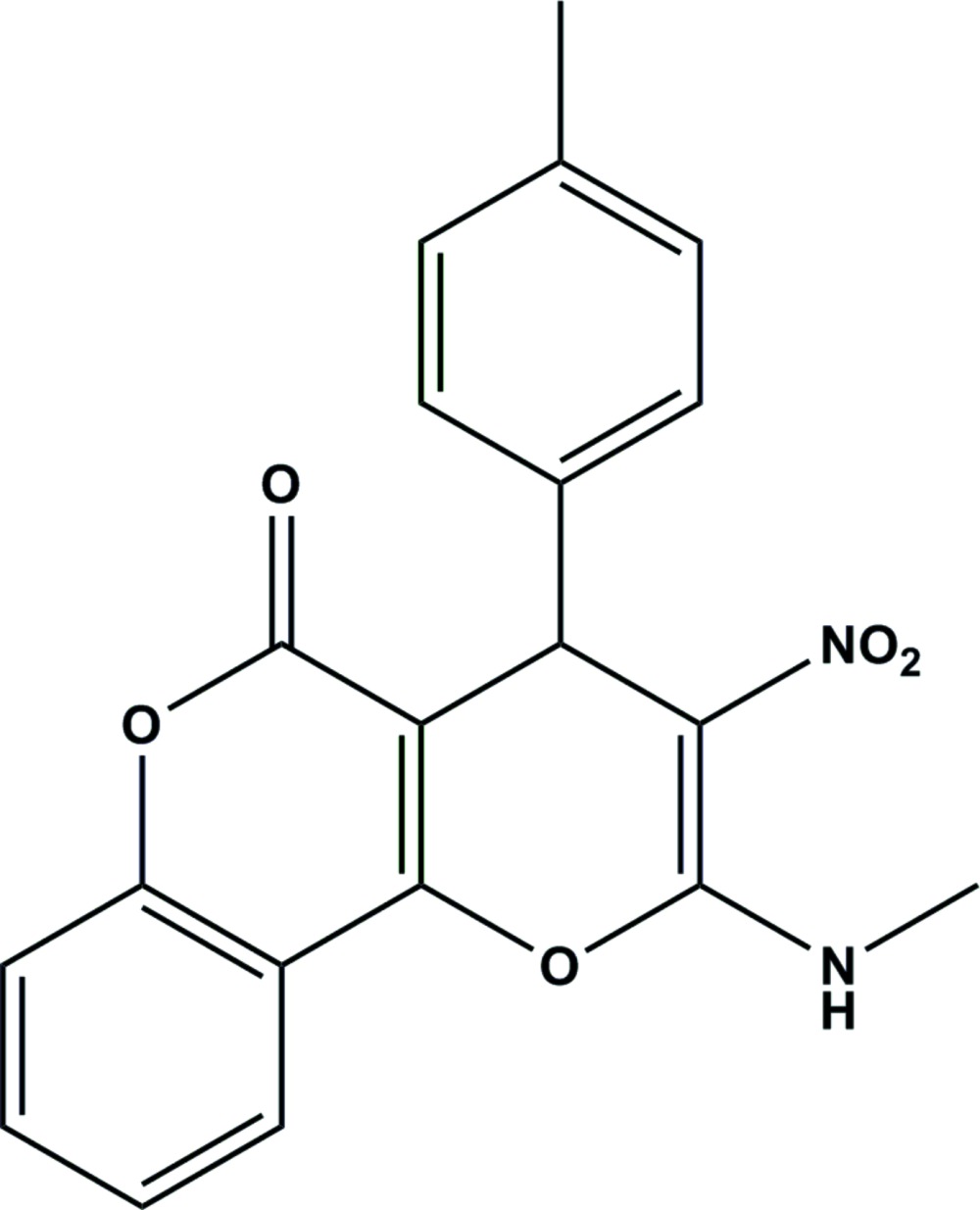


## Experimental   

### Crystal data  

C_20_H_16_N_2_O_5_
*M* *_r_* = 364.35
Monoclinic, 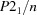

*a* = 10.8336 (11) Å
*b* = 11.7927 (11) Å
*c* = 13.7275 (14) Å
β = 108.357 (2)°
*V* = 1664.5 (3) Å^3^
*Z* = 4
Mo *K*α radiation
μ = 0.11 mm^−1^
*T* = 293 K
0.21 × 0.19 × 0.18 mm

### Data collection  

Bruker SMART APEXII CCD diffractometer
Absorption correction: multi-scan (*SADABS*; Bruker, 2008 ▸) *T*_min_ = 0.978, *T*_max_ = 0.981
28712 measured reflections
4571 independent reflections
2862 reflections with *I* > 2σ(*I*)
*R*_int_ = 0.039

### Refinement  

*R*[*F* ^2^ > 2σ(*F* ^2^)] = 0.050
*wR*(*F* ^2^) = 0.151
*S* = 1.03
4571 reflections
244 parameters
H-atom parameters constrained
Δρ_max_ = 0.26 e Å^−3^
Δρ_min_ = −0.36 e Å^−3^

### 

Data collection: *APEX2* (Bruker, 2008 ▸); cell refinement: *SAINT* (Bruker, 2008 ▸); data reduction: *SAINT*; program(s) used to solve structure: *SHELXS97* (Sheldrick, 2008 ▸); program(s) used to refine structure: *SHELXL97* (Sheldrick, 2008 ▸); molecular graphics: *ORTEP-3 for Windows* (Farrugia, 2012 ▸); software used to prepare material for publication: *SHELXL97* and *PLATON* (Spek, 2009 ▸).

## Supplementary Material

Crystal structure: contains datablock(s) global, I. DOI: 10.1107/S205698901500225X/hb7345sup1.cif

Structure factors: contains datablock(s) I. DOI: 10.1107/S205698901500225X/hb7345Isup2.hkl

Supporting information file. DOI: 10.1107/S205698901500225X/hb7345Isup3.cml

**Fig. 1.**
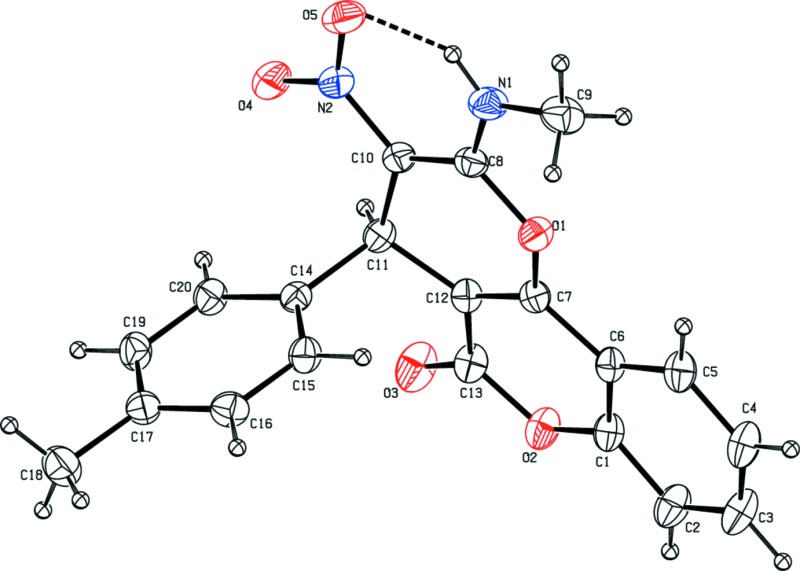
The molecular structure of the title molecule, with atom displacement ellipsoids drawn at the 30% probability level. The intramolecular N—H···O hydrogen bond, which generates an S(6) ring motif, is shown as a dashed line.

**Fig. 2.**
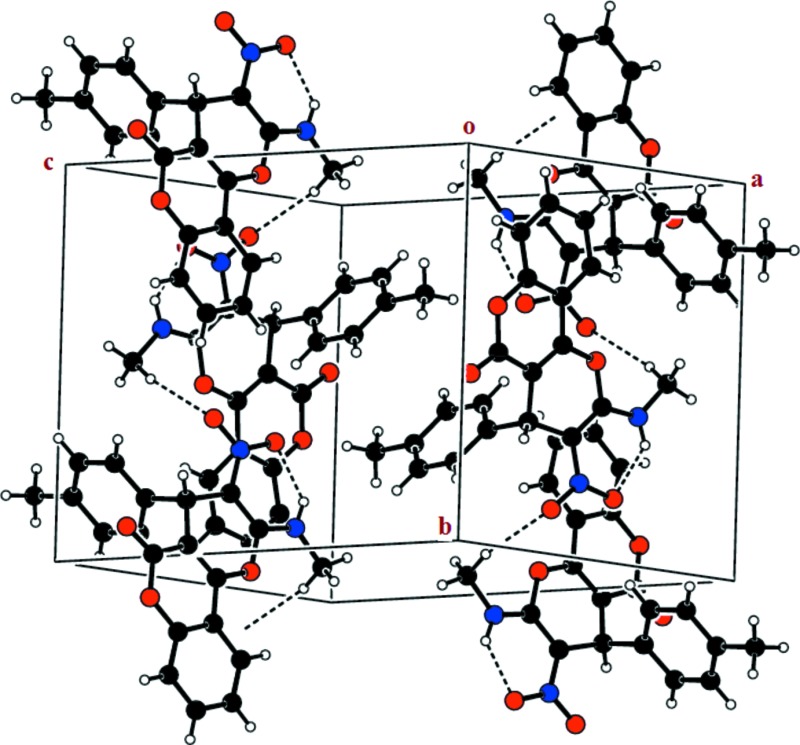
The crystal packing of the title compound, viewed along the b axis, showing C9—H9C···O4 hydrogen bonds producing chains parallel to the 101 planes.

. DOI: 10.1107/S205698901500225X/hb7345fig1.tif
The mol­ecular structure of the title mol­ecule, with atom displacement ellipsoids drawn at the 30% probability level. The intra­molecular N—H⋯O hydrogen bond, which generates an S(6) ring motif, is shown as a dashed line.

b C . DOI: 10.1107/S205698901500225X/hb7345fig2.tif
The crystal packing of the title compound, viewed along the *b* axis, showing C9—H9*C*⋯O4 hydrogen bonds producing chains parallel to the 101 planes.

CCDC reference: 1046918

Additional supporting information:  crystallographic information; 3D view; checkCIF report

## Figures and Tables

**Table 1:** Hydrogen-bond geometry (, )

*D*H*A*	*D*H	H*A*	*D* *A*	*D*H*A*
N1H1O5	0.86	1.96	2.590(2)	129
C9H9*C*O4^i^	0.96	2.46	3.389(3)	163

Symmetry code: (i) 
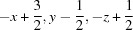
.

## supplementary crystallographic information

### S1. Comment

Coumarins and their natural synthetic derivatives are pharmacologically interesting compounds due to their structural diversity and synthetic accessibility (Borges *et al.*, 2005, 2009). Cancer, a diverse group of diseases characterized by uncontrolled growth of abnormal cells, is a major worldwide problem. It is a fatal disease standing next to the cardiovascular disease in terms of morbidity and mortality. Although the cancer research has led to a number of new and effective solutions, the medicines used as treatments have clear limitations and unfortunately cancer is projected as the primary cause of death in the future(Gibbs *et al.* 2000; Varmus *et al.* 2006).

The title compound, Fig. 1, consists of a chromene moiety attached to a nitrophenyl ring, a nitro group and a methylamine group. The molecular structure is stabilized by an intra molecular N1—H1A···O5 interaction, which generates an S(6) ring motif. The chromen ring is almost coplanar with the least-square planes of the phenyl ring, making dihedral angle of 87.18 (8)°. The six-membered pyran ring(C7/O1/C8/C10/C11/C12) adopts sofa conformatons, with puckering parameters Q_2_ = 0.1787 (17) Å, Q_3_ = -0.0688 (18) Å and φ_2_ = 0.9 (6)°, respectively. The C18 atom deviates from mean plane of the phenyl ring by -0.0260 Å. The title compound exihibits structural similarities with an already reported related structure (Narayanan *et al.* 2013).

In the crystal, the molecules are linked *via* intermolecular C9—H9C···O4 hydrogen-bond interaction to generate [010] chains.

### S2. Experimental

A solution of 4-methylbenzaldehyde (1.0 mmol), 4-hydroxycoumarin (1.0 mmol), NMSM (1.0 mmol), and piperidine (0.2 equiv) in EtOH (2 ml) was stirred for the three hours. After reaction was complete as indicated by TLC, the product was filtered and washed with EtOH (2 ml) to remove the excess base and other impurities. Finally, the product was recrystallized from EtOH to yield colourless blocks of the title compound.

### S3. Refinement

N and C-bound H atoms were positioned geometrically (C–H = 0.93–0.98 Å) and allowed to ride on their parent atoms, with *U*_iso_(H) = 1.5*U*_eq_(C) for methyl H atoms and 1.2*U*_eq_(C) for all other H atoms.

### Crystal data

**Table:**

C_20_H_16_N_2_O_5_	*F*(000) = 760
*M**_r_* = 364.35	*D*_x_ = 1.454 Mg m^−^^3^
Monoclinic, *P*2_1_/*n*	Mo *K*α radiation, λ = 0.71073 Å
Hall symbol: -P 2yn	Cell parameters from 2862 reflections
*a* = 10.8336 (11) Å	θ = 2.1–30.3°
*b* = 11.7927 (11) Å	µ = 0.11 mm^−^^1^
*c* = 13.7275 (14) Å	*T* = 293 K
β = 108.357 (2)°	Block, colourless
*V* = 1664.5 (3) Å^3^	0.21 × 0.19 × 0.18 mm
*Z* = 4	

### Data collection

**Table:**

Bruker SMART APEXII CCD diffractometer	4571 independent reflections
Radiation source: fine-focus sealed tube	2862 reflections with *I* > 2σ(*I*)
Graphite monochromator	*R*_int_ = 0.039
ω and φ scans	θ_max_ = 30.3°, θ_min_ = 2.1°
Absorption correction: multi-scan (*SADABS*; Bruker, 2008)	*h* = −15→15
*T*_min_ = 0.978, *T*_max_ = 0.981	*k* = −15→16
28712 measured reflections	*l* = −19→19

### Refinement

**Table:**

Refinement on *F*^2^	Primary atom site location: structure-invariant direct methods
Least-squares matrix: full	Secondary atom site location: difference Fourier map
*R*[*F*^2^ > 2σ(*F*^2^)] = 0.050	Hydrogen site location: inferred from neighbouring sites
*wR*(*F*^2^) = 0.151	H-atom parameters constrained
*S* = 1.03	*w* = 1/[σ^2^(*F*_o_^2^) + (0.0607*P*)^2^ + 0.7266*P*] where *P* = (*F*_o_^2^ + 2*F*_c_^2^)/3
4571 reflections	(Δ/σ)_max_ = 0.001
244 parameters	Δρ_max_ = 0.26 e Å^−^^3^
0 restraints	Δρ_min_ = −0.36 e Å^−^^3^

### Special details

**Table:**

Geometry. All e.s.d.'s (except the e.s.d. in the dihedral angle between two l.s. planes) are estimated using the full covariance matrix. The cell e.s.d.'s are taken into account individually in the estimation of e.s.d.'s in distances, angles and torsion angles; correlations between e.s.d.'s in cell parameters are only used when they are defined by crystal symmetry. An approximate (isotropic) treatment of cell e.s.d.'s is used for estimating e.s.d.'s involving l.s. planes.
Refinement. Refinement of *F*^2^ against ALL reflections. The weighted *R*-factor *wR* and goodness of fit *S* are based on *F*^2^, conventional *R*-factors *R* are based on *F*, with *F* set to zero for negative *F*^2^. The threshold expression of *F*^2^ > σ(*F*^2^) is used only for calculating *R*-factors(gt) *etc*. and is not relevant to the choice of reflections for refinement. *R*-factors based on *F*^2^ are statistically about twice as large as those based on *F*, and *R*- factors based on ALL data will be even larger.

### Fractional atomic coordinates and isotropic or equivalent isotropic displacement parameters (Å^2^)

**Table:**

	*x*	*y*	*z*	*U*_iso_*/*U*_eq_	
C1	0.39731 (17)	0.29273 (15)	0.11931 (14)	0.0394 (4)	
C2	0.3614 (2)	0.18037 (17)	0.11445 (17)	0.0525 (5)	
H2	0.2802	0.1594	0.1181	0.063*	
C3	0.4481 (2)	0.09986 (18)	0.10411 (18)	0.0569 (6)	
H3	0.4256	0.0235	0.1014	0.068*	
C4	0.5677 (2)	0.13063 (17)	0.09769 (17)	0.0538 (5)	
H4	0.6246	0.0751	0.0895	0.065*	
C5	0.60377 (19)	0.24263 (17)	0.10328 (15)	0.0455 (4)	
H5	0.6849	0.2629	0.0990	0.055*	
C6	0.51827 (16)	0.32599 (14)	0.11533 (13)	0.0358 (4)	
C7	0.54773 (16)	0.44453 (14)	0.13040 (13)	0.0342 (4)	
C8	0.70721 (17)	0.58447 (15)	0.13873 (13)	0.0375 (4)	
C9	0.9063 (2)	0.5072 (2)	0.11560 (18)	0.0560 (5)	
H9A	0.9850	0.5386	0.1089	0.084*	
H9B	0.8626	0.4640	0.0553	0.084*	
H9C	0.9270	0.4587	0.1747	0.084*	
C10	0.62572 (17)	0.66705 (14)	0.15559 (13)	0.0365 (4)	
C11	0.50320 (16)	0.64000 (14)	0.18012 (13)	0.0359 (4)	
H11	0.4340	0.6918	0.1422	0.043*	
C12	0.46442 (16)	0.52058 (15)	0.14680 (13)	0.0350 (4)	
C13	0.33590 (17)	0.48409 (16)	0.14373 (14)	0.0422 (4)	
C14	0.52303 (15)	0.65125 (14)	0.29503 (13)	0.0331 (4)	
C15	0.60210 (17)	0.57475 (15)	0.36318 (14)	0.0400 (4)	
H15	0.6410	0.5159	0.3384	0.048*	
C16	0.62389 (18)	0.58497 (16)	0.46753 (15)	0.0445 (4)	
H16	0.6773	0.5328	0.5120	0.053*	
C17	0.56764 (17)	0.67147 (16)	0.50698 (14)	0.0414 (4)	
C18	0.5931 (2)	0.6825 (2)	0.62041 (16)	0.0597 (6)	
H18A	0.6515	0.6236	0.6554	0.090*	
H18B	0.5127	0.6760	0.6354	0.090*	
H18C	0.6316	0.7552	0.6431	0.090*	
C19	0.48681 (18)	0.74572 (16)	0.43833 (15)	0.0449 (4)	
H19	0.4466	0.8037	0.4630	0.054*	
C20	0.46409 (17)	0.73612 (15)	0.33388 (14)	0.0405 (4)	
H20	0.4087	0.7872	0.2894	0.049*	
N1	0.82208 (16)	0.59828 (15)	0.12756 (13)	0.0480 (4)	
H1	0.8497	0.6666	0.1273	0.058*	
N2	0.66129 (17)	0.78014 (13)	0.15862 (12)	0.0455 (4)	
O1	0.67089 (11)	0.47326 (10)	0.13148 (10)	0.0405 (3)	
O2	0.30759 (12)	0.37082 (11)	0.12920 (11)	0.0469 (3)	
O3	0.25155 (13)	0.54490 (13)	0.15308 (14)	0.0624 (4)	
O4	0.58643 (16)	0.85218 (12)	0.17480 (12)	0.0591 (4)	
O5	0.76697 (15)	0.80934 (12)	0.14575 (12)	0.0598 (4)	

### Atomic displacement parameters (Å^2^)

**Table:**

	*U*^11^	*U*^22^	*U*^33^	*U*^12^	*U*^13^	*U*^23^
C1	0.0360 (9)	0.0392 (9)	0.0391 (9)	0.0006 (7)	0.0063 (7)	−0.0084 (7)
C2	0.0418 (10)	0.0437 (11)	0.0668 (14)	−0.0075 (8)	0.0098 (9)	−0.0126 (10)
C3	0.0534 (12)	0.0367 (10)	0.0722 (15)	−0.0048 (9)	0.0077 (10)	−0.0144 (10)
C4	0.0517 (12)	0.0410 (11)	0.0642 (13)	0.0066 (9)	0.0117 (10)	−0.0147 (9)
C5	0.0421 (10)	0.0443 (11)	0.0492 (11)	0.0049 (8)	0.0129 (8)	−0.0096 (8)
C6	0.0367 (9)	0.0356 (9)	0.0325 (9)	0.0017 (7)	0.0073 (7)	−0.0051 (7)
C7	0.0324 (8)	0.0367 (9)	0.0324 (8)	0.0011 (7)	0.0089 (7)	−0.0012 (7)
C8	0.0418 (9)	0.0384 (9)	0.0338 (9)	0.0000 (7)	0.0140 (7)	0.0048 (7)
C9	0.0470 (11)	0.0626 (13)	0.0669 (14)	0.0091 (10)	0.0301 (10)	0.0089 (11)
C10	0.0412 (9)	0.0320 (8)	0.0374 (9)	0.0011 (7)	0.0139 (7)	0.0033 (7)
C11	0.0342 (8)	0.0303 (8)	0.0417 (9)	0.0059 (7)	0.0097 (7)	0.0013 (7)
C12	0.0332 (8)	0.0357 (9)	0.0342 (9)	0.0034 (7)	0.0079 (7)	−0.0016 (7)
C13	0.0346 (9)	0.0415 (10)	0.0470 (10)	0.0018 (7)	0.0081 (8)	−0.0061 (8)
C14	0.0298 (8)	0.0308 (8)	0.0402 (9)	−0.0002 (6)	0.0131 (7)	−0.0001 (7)
C15	0.0393 (9)	0.0360 (9)	0.0457 (10)	0.0082 (7)	0.0149 (8)	−0.0012 (7)
C16	0.0436 (10)	0.0439 (10)	0.0438 (10)	0.0067 (8)	0.0107 (8)	0.0084 (8)
C17	0.0385 (9)	0.0442 (10)	0.0441 (10)	−0.0077 (8)	0.0165 (8)	−0.0041 (8)
C18	0.0679 (14)	0.0687 (15)	0.0450 (12)	−0.0062 (11)	0.0212 (10)	−0.0041 (10)
C19	0.0460 (10)	0.0415 (10)	0.0517 (11)	0.0046 (8)	0.0220 (9)	−0.0075 (8)
C20	0.0385 (9)	0.0366 (9)	0.0466 (10)	0.0071 (7)	0.0138 (8)	−0.0001 (8)
N1	0.0472 (9)	0.0462 (9)	0.0595 (10)	−0.0008 (7)	0.0295 (8)	0.0051 (8)
N2	0.0586 (10)	0.0366 (8)	0.0424 (9)	−0.0015 (7)	0.0176 (8)	0.0045 (7)
O1	0.0364 (6)	0.0360 (7)	0.0525 (8)	0.0021 (5)	0.0189 (6)	−0.0005 (6)
O2	0.0344 (6)	0.0419 (7)	0.0626 (9)	−0.0018 (5)	0.0129 (6)	−0.0120 (6)
O3	0.0376 (7)	0.0541 (9)	0.0952 (12)	0.0077 (6)	0.0204 (8)	−0.0142 (8)
O4	0.0780 (10)	0.0349 (7)	0.0708 (10)	0.0064 (7)	0.0328 (8)	0.0035 (7)
O5	0.0682 (10)	0.0461 (8)	0.0738 (11)	−0.0111 (7)	0.0348 (8)	0.0063 (7)

### Geometric parameters (Å, º)

**Table:**

C1—O2	1.376 (2)	C11—C12	1.499 (2)
C1—C2	1.377 (3)	C11—C14	1.529 (2)
C1—C6	1.385 (2)	C11—H11	0.9800
C2—C3	1.374 (3)	C12—C13	1.445 (2)
C2—H2	0.9300	C13—O3	1.200 (2)
C3—C4	1.374 (3)	C13—O2	1.371 (2)
C3—H3	0.9300	C14—C20	1.381 (2)
C4—C5	1.373 (3)	C14—C15	1.385 (2)
C4—H4	0.9300	C15—C16	1.382 (3)
C5—C6	1.396 (2)	C15—H15	0.9300
C5—H5	0.9300	C16—C17	1.383 (3)
C6—C7	1.434 (2)	C16—H16	0.9300
C7—C12	1.341 (2)	C17—C19	1.379 (3)
C7—O1	1.372 (2)	C17—C18	1.499 (3)
C8—N1	1.311 (2)	C18—H18A	0.9600
C8—O1	1.364 (2)	C18—H18B	0.9600
C8—C10	1.382 (2)	C18—H18C	0.9600
C9—N1	1.452 (3)	C19—C20	1.381 (3)
C9—H9A	0.9600	C19—H19	0.9300
C9—H9B	0.9600	C20—H20	0.9300
C9—H9C	0.9600	N1—H1	0.8600
C10—N2	1.385 (2)	N2—O4	1.242 (2)
C10—C11	1.503 (2)	N2—O5	1.260 (2)
			
O2—C1—C2	116.81 (17)	C7—C12—C13	119.29 (16)
O2—C1—C6	121.38 (16)	C7—C12—C11	122.74 (15)
C2—C1—C6	121.81 (17)	C13—C12—C11	117.61 (15)
C3—C2—C1	118.54 (19)	O3—C13—O2	117.06 (17)
C3—C2—H2	120.7	O3—C13—C12	125.32 (18)
C1—C2—H2	120.7	O2—C13—C12	117.62 (15)
C2—C3—C4	120.87 (19)	C20—C14—C15	118.29 (16)
C2—C3—H3	119.6	C20—C14—C11	121.95 (15)
C4—C3—H3	119.6	C15—C14—C11	119.76 (15)
C5—C4—C3	120.53 (19)	C16—C15—C14	120.72 (16)
C5—C4—H4	119.7	C16—C15—H15	119.6
C3—C4—H4	119.7	C14—C15—H15	119.6
C4—C5—C6	119.76 (19)	C15—C16—C17	121.18 (17)
C4—C5—H5	120.1	C15—C16—H16	119.4
C6—C5—H5	120.1	C17—C16—H16	119.4
C1—C6—C5	118.46 (17)	C19—C17—C16	117.64 (17)
C1—C6—C7	116.14 (15)	C19—C17—C18	121.61 (18)
C5—C6—C7	125.29 (17)	C16—C17—C18	120.75 (19)
C12—C7—O1	122.46 (16)	C17—C18—H18A	109.5
C12—C7—C6	123.04 (16)	C17—C18—H18B	109.5
O1—C7—C6	114.43 (14)	H18A—C18—H18B	109.5
N1—C8—O1	111.93 (16)	C17—C18—H18C	109.5
N1—C8—C10	127.74 (17)	H18A—C18—H18C	109.5
O1—C8—C10	120.33 (15)	H18B—C18—H18C	109.5
N1—C9—H9A	109.5	C17—C19—C20	121.64 (17)
N1—C9—H9B	109.5	C17—C19—H19	119.2
H9A—C9—H9B	109.5	C20—C19—H19	119.2
N1—C9—H9C	109.5	C19—C20—C14	120.49 (17)
H9A—C9—H9C	109.5	C19—C20—H20	119.8
H9B—C9—H9C	109.5	C14—C20—H20	119.8
C8—C10—N2	119.80 (16)	C8—N1—C9	125.09 (17)
C8—C10—C11	122.95 (15)	C8—N1—H1	117.5
N2—C10—C11	117.04 (15)	C9—N1—H1	117.5
C12—C11—C10	108.26 (14)	O4—N2—O5	120.76 (16)
C12—C11—C14	109.33 (14)	O4—N2—C10	118.21 (16)
C10—C11—C14	111.45 (14)	O5—N2—C10	121.03 (16)
C12—C11—H11	109.3	C8—O1—C7	119.69 (13)
C10—C11—H11	109.3	C13—O2—C1	122.25 (14)
C14—C11—H11	109.3		
			
O2—C1—C2—C3	179.72 (18)	C11—C12—C13—O3	9.9 (3)
C6—C1—C2—C3	−0.8 (3)	C7—C12—C13—O2	3.3 (3)
C1—C2—C3—C4	−0.6 (3)	C11—C12—C13—O2	−169.93 (15)
C2—C3—C4—C5	1.1 (3)	C12—C11—C14—C20	−129.00 (17)
C3—C4—C5—C6	−0.2 (3)	C10—C11—C14—C20	111.35 (18)
O2—C1—C6—C5	−178.80 (16)	C12—C11—C14—C15	51.0 (2)
C2—C1—C6—C5	1.7 (3)	C10—C11—C14—C15	−68.7 (2)
O2—C1—C6—C7	4.9 (2)	C20—C14—C15—C16	−1.6 (3)
C2—C1—C6—C7	−174.59 (18)	C11—C14—C15—C16	178.42 (16)
C4—C5—C6—C1	−1.2 (3)	C14—C15—C16—C17	0.0 (3)
C4—C5—C6—C7	174.73 (18)	C15—C16—C17—C19	1.4 (3)
C1—C6—C7—C12	−0.5 (3)	C15—C16—C17—C18	−179.30 (18)
C5—C6—C7—C12	−176.51 (18)	C16—C17—C19—C20	−1.2 (3)
C1—C6—C7—O1	176.64 (15)	C18—C17—C19—C20	179.47 (18)
C5—C6—C7—O1	0.6 (3)	C17—C19—C20—C14	−0.4 (3)
N1—C8—C10—N2	2.4 (3)	C15—C14—C20—C19	1.8 (3)
O1—C8—C10—N2	−177.36 (15)	C11—C14—C20—C19	−178.26 (16)
N1—C8—C10—C11	−172.24 (17)	O1—C8—N1—C9	−3.8 (3)
O1—C8—C10—C11	8.0 (3)	C10—C8—N1—C9	176.42 (19)
C8—C10—C11—C12	−19.5 (2)	C8—C10—N2—O4	−179.39 (17)
N2—C10—C11—C12	165.70 (15)	C11—C10—N2—O4	−4.5 (2)
C8—C10—C11—C14	100.73 (19)	C8—C10—N2—O5	0.3 (3)
N2—C10—C11—C14	−74.03 (19)	C11—C10—N2—O5	175.25 (16)
O1—C7—C12—C13	179.56 (15)	N1—C8—O1—C7	−173.09 (15)
C6—C7—C12—C13	−3.6 (3)	C10—C8—O1—C7	6.7 (2)
O1—C7—C12—C11	−7.6 (3)	C12—C7—O1—C8	−7.0 (2)
C6—C7—C12—C11	169.29 (16)	C6—C7—O1—C8	175.86 (15)
C10—C11—C12—C7	19.3 (2)	O3—C13—O2—C1	−178.85 (17)
C14—C11—C12—C7	−102.29 (19)	C12—C13—O2—C1	1.0 (3)
C10—C11—C12—C13	−167.72 (15)	C2—C1—O2—C13	174.23 (17)
C14—C11—C12—C13	70.70 (19)	C6—C1—O2—C13	−5.2 (3)
C7—C12—C13—O3	−176.84 (19)		

### Hydrogen-bond geometry (Å, º)

**Table:**

*D*—H···*A*	*D*—H	H···*A*	*D*···*A*	*D*—H···*A*
N1—H1···O5	0.86	1.96	2.590 (2)	129
C9—H9*C*···O4^i^	0.96	2.46	3.389 (3)	163

Symmetry code: (i) −*x*+3/2, *y*−1/2, −*z*+1/2.
