# Supplementary figures and images for: Crystal structure of 2-methyl­amino-3-nitro-4-p-tolyl­pyrano[3,2-c]chromen-5(4H)-one
Source: Acta Crystallogr E Crystallogr Commun. 2015 Feb 7;71(Pt 3):o158–9. doi: 10.1107/S205698901500225X (PMC4350753; doi:10.1107/S205698901500225X)

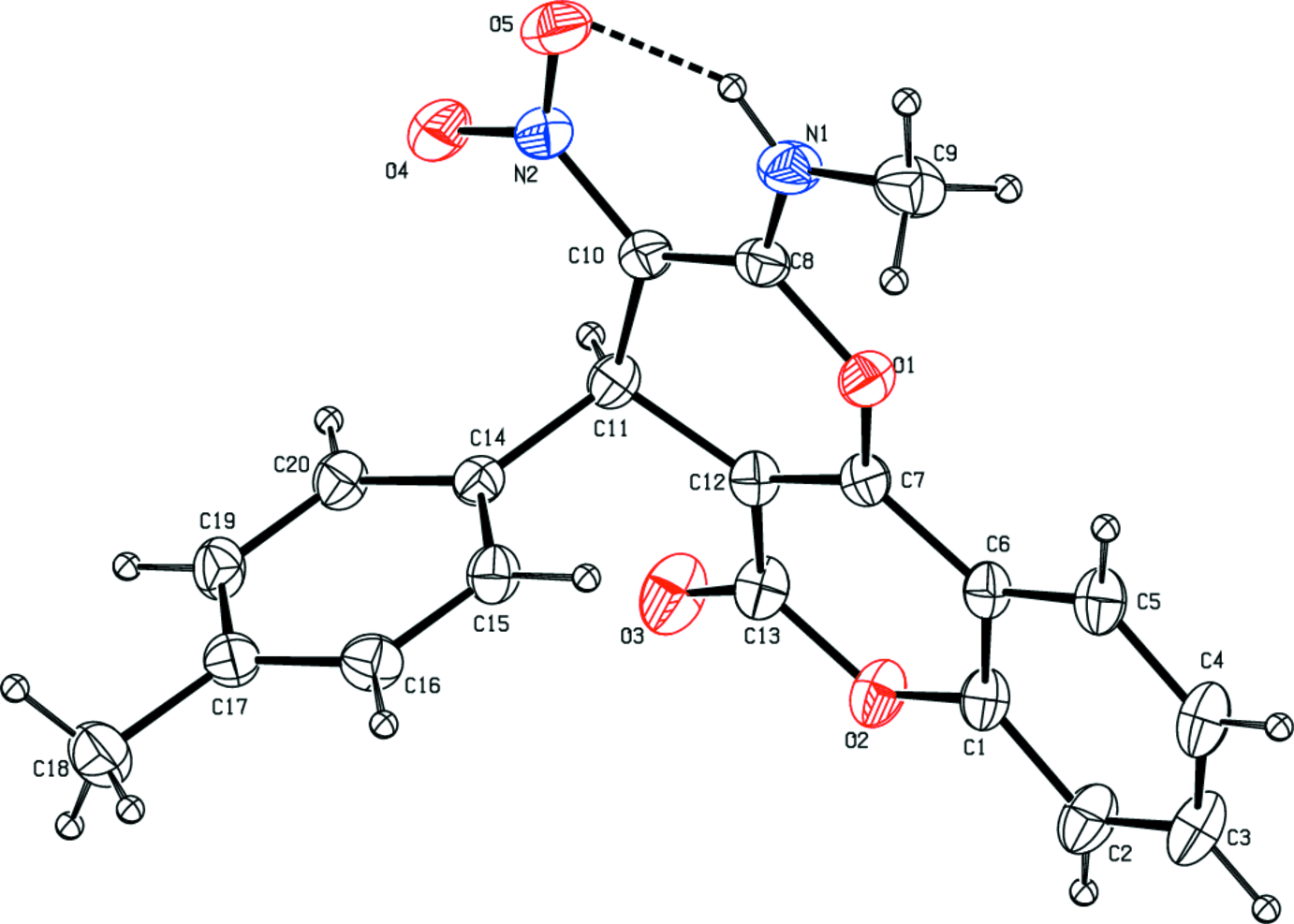

Supplement: Supplementary file 4 [file e-71-0o158-fig1.tif]

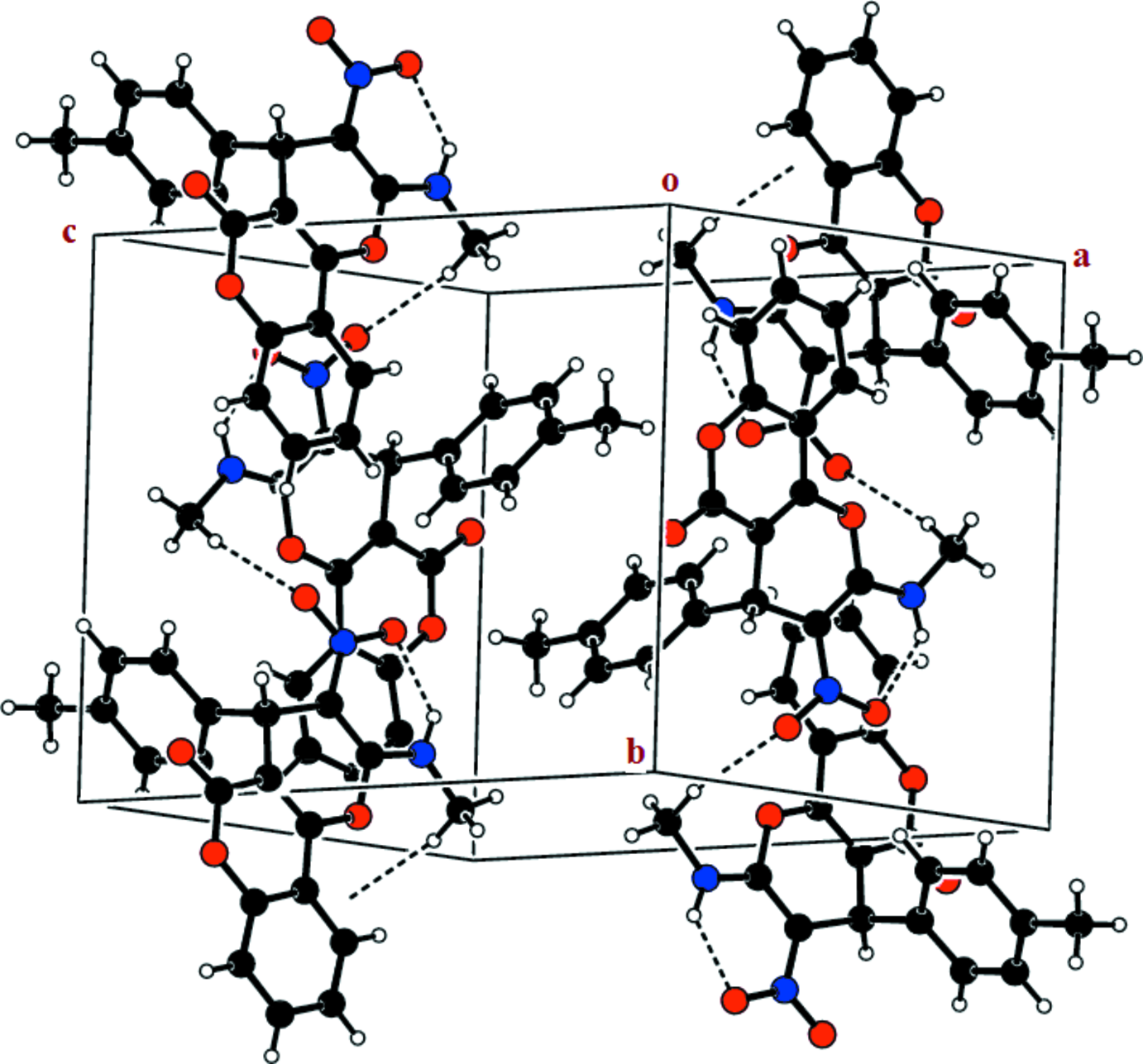

Supplement: Supplementary file 5 [file e-71-0o158-fig2.tif]
